# Extracorporeal photopheresis for pembrolizumab-induced dermatitis: a case report

**DOI:** 10.1093/skinhd/vzaf045

**Published:** 2025-06-25

**Authors:** Veronika Zenderowski, James A Hutchinson, Andreas Brosig, Sebastian Haferkamp, Katharina Kronenberg

**Affiliations:** Department of Dermatology, University Hospital Regensburg, Regensburg, Germany; Department of Surgery, University Hospital Regensburg, Regensburg, Germany; Institute for Clinical Chemistry and Laboratory Medicine, University Hospital Regensburg, Regensburg, Germany; Department of Dermatology, University Hospital Regensburg, Regensburg, Germany; Department of Surgery, University Hospital Regensburg, Regensburg, Germany

## Abstract

Immune-related adverse events (irAE) are common in checkpoint blockade–treated patients and limit its clinical application. Corticosteroids are the first-line therapy for treatment of irAE, but animal models clearly demonstrate that steroids diminish anti-programmed cell death protein 1 (PD-1)-induced tumour immunity. Better strategies to manage irAE while preserving anti-tumour immunity are needed. Extracorporeal photopheresis (ECP) was recently introduced as second-line treatment for steroid-refractory immune checkpoint inhibitor (ICI)-related colitis and hepatitis. Here, we extend the application of ECP to immune-related maculopapular rash after adjuvant anti-PD-1 therapy in a single melanoma patient. The patient’s dermatitis markedly improved after off-label ECP, with a substantial reduction in skin lesions and pruritus scores, and stabilization of immune markers. The patient remained well after ECP with no recurrent or metastatic disease at 14 months after starting ECP treatment. Hence, in this case, ECP led to successful resolution of pembrolizumab-induced dermatitis and a favourable oncological outcome.

## Teaser

Treatment of immune checkpoint inhibitor–related adverse reactions with extracorporeal phoropheresis (ECP) has become a hot topic. Here, we extend the application of ECP from colitis, hepatitis and pneumonitis to a case of treatment-refractory immune-related maculopapular rash after adjuvant anti-PD-1 therapy. Under ECP, there was a rapid subjective and objective in the patient’s dermatitis, as well as stabilization of immune markers. Importantly, the patient remained well after ECP with no recurrent or metastatic disease at 14 months.

**What is already known about this topic?**

- Corticosteroids are first-line therapy for immune checkpoint inhibitor (ICI)-related adverse events.
- Mouse models demonstrate that steroids are detrimental to anti-programmed cell death receptor 1 (PD-1)-induced tumour immunity.
- Extracorporeal photopheresis (ECP) has shown promise as second-line therapy for ICI-related colitis and hepatitis.

**What does this study add?**

- Here, we successfully apply ECP in a case of steroid-refractory ICI-related dermatitis.

Although serious immune-related adverse events (irAE) after immune checkpoint inhibitor (ICI) therapy for melanoma are infrequent, they invariably disrupt treatment and may require immunosuppression – conventionally, corticosteroids as first-line, escalating to mycophenolate mofetil, azathioprine, infliximab [α-tumour necrosis factor (TNF)] or vedolizumab (α-integrin α_4_β_7_). Whether steroids limit clinical efficacy of ICI is controversial and possibly depends upon timing;^1,2^ however, mouse models clearly demonstrate that prednisolone, α-TNF and α-α_4_β_7_ are detrimental in α-programmed cell death receptor 1 (PD-1)-induced tumour immunity.^3^ Concerns about irAE risk influence the decision to prescribe adjuvant ICI in stage II melanoma patients, despite KEYNOTE-716 and Checkmate 76 registering improved recurrence-free survival and distant metastasis-free survival.^4,5^ Consequently, there is an urgent need for better strategies to manage irAE caused by α-PD-1 in adjuvant settings, which can control adverse reactions while preserving anti-tumour immunity.^6^

Extracorporeal photopheresis (ECP) is a safe therapy with long-established applications in various T-cell mediated diseases.^7^ The ECP procedure involves collecting autologous peripheral blood leucocytes, which are driven into apoptosis before being reinfused intravenously. ECP acts primarily through *in situ* exposure of mononuclear phagocytes to apoptotic cells, which then suppress inflammation, promote specific regulatory T-cell responses and retard fibrosis.^8,9^ Because different populations of macrophages and dendritic cells (DC) play diverse, often contradictory roles in immune pathologies, the actions of ECP are context specific. In experimental ICI-induced colitis, the critical action of ECP appears to be reprogramming of tissue macrophages after efferocytosis of apoptotic cells from photopheresates, which then locally suppress inflammation. Accumulating clinical evidence from case reports and a small phase 1b/2 trial supports ECP as a second-line therapy for irAE, especially colitis or hepatitis.^3,10^

## Case report

**Figure 1:**
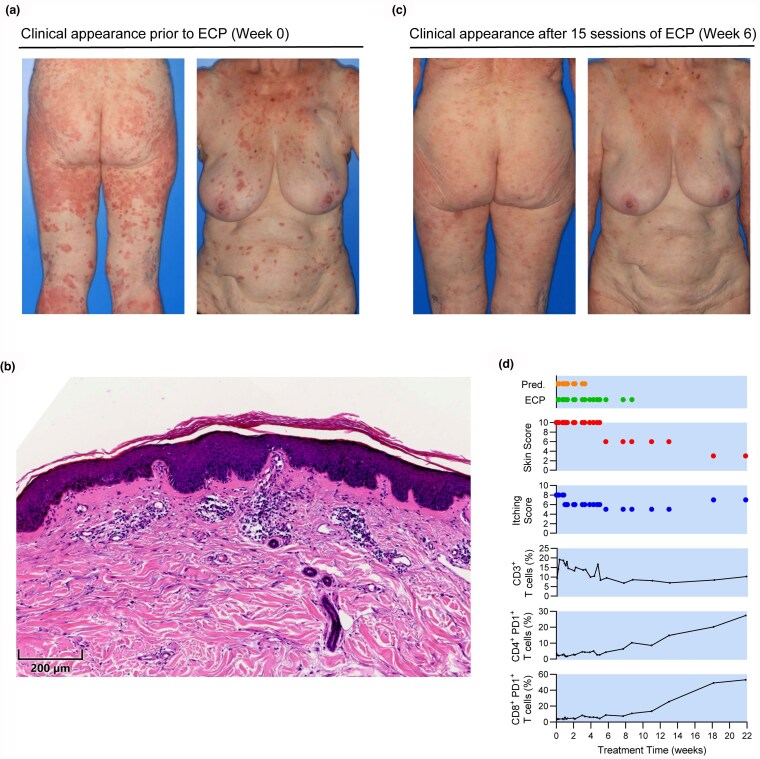
Extracorporeal photopheresis (ECP) treatment of steroid-refractory, Common Terminology Criteria for Adverse Events (CTCAE) grade III dermatitis caused by adjuvant pembrolizumab therapy in an 83-year-old patient with stage IIB melanoma. (a) Clinical appearance prior to ECP. (b) Haematoxylin and eosin (H&E) staining of lesional skin biopsy pre-ECP showing an unremarkable epidermis. The upper dermis reveals moderately pronounced lymphocytic infiltrates, increased plasma cells and subepidermal edema, consistent with non-specific dermatitis. (c) Clinical appearance after 6 weeks of ECP. (d) Improvement in skin lesions and symptoms allowed weaning of prednisolone (Pred.) within 4 weeks of starting ECP. Peripheral T cell subset distributions were monitored with established methods.^20^ Notably, clinical improvement coincided with a stabilization of peripheral T cell frequencies and preceded pembrolizumab clearance.

An 83-year-old woman presenting with cutaneous melanoma on the right temple (depth 6 mm, stage IIB) underwent wide local excision and sentinel lymph node biopsy. Histopathological examination and full-body computed tomography (CT) imaging – from the skull to the pelvis – showed no signs of metastatic disease. Adjuvant immunotherapy with 400 mg pembrolizumab at 6-week intervals was initially well-tolerated. After the sixth dose, pruritic skin lesions developed over her entire body that were refractory to topical and systemic corticosteroids; accordingly, pembrolizumab was stopped after eight doses. At this time, the patient exhibited multiple, disseminated erythematous macules and papules across the trunk and upper extremities. Lesions on the upper back had a verrucous appearance, whereas those on the lower legs and thighs were erythematous plaques (Figure 1a). A sharply demarcated erythema extended across the nose and cheeks. Notably, there was no mucosal involvement. The patient reported severe pruritus, rated 8 out of 10 on the Numerical Rating Scale. Excluding other causes, a clinical and histopathological diagnosis of immune-related maculopapular rash [Common Terminology Criteria for Adverse Events (CTCAE) grade 3] was made. Histopathological examination showed moderate lymphocytic inflammatory infiltrates, an increased number of plasma cells and subepidermal edema (Figure 1b). Initial treatment included topical corticosteroids, oral antihistamines and polidocanol-containing topical preparations.

Owing to her dermatitis, the patient was hospitalized twice for high-dose prednisolone therapy. Initially, she received 50 mg daily (body weight: 60 kg), tapered by 10 mg every 3 days. During the second admission, treatment was intensified to 70 mg daily with tapering every 3–5 days. However, this regimen failed to improve her symptoms. A decision was made to commence off-label ECP treatment, beginning with an intensive phase of six sessions over 2 weeks and twice-weekly sessions thereafter. ECP was initially performed using a Sheldon catheter. After 2 weeks, treatment was continued in an outpatient setting using peripheral venous access. Following marked improvement, oral prednisolone was stopped by 4 weeks and ECP was reduced to once-weekly sessions. ECP was terminated at 9 weeks with a substantial reduction in skin lesion and pruritus scores (Figure 1c, d). Immune monitoring revealed a rapid stabilization of peripheral blood T cell frequency after 4–5 weeks of ECP that coincided with clinical improvement. Notably, skin lesions and itching began to improve before pembrolizumab was cleared, as shown by the increasing frequency of PD-1^+^ T cells after 8 weeks. This suggests that the drug was still bound to PD-1 receptors and that clinical improvement occurred despite its continued presence in the system.

CT imaging during ECP showed no detectable metastases. A follow-up PET-CT scan at 14 months after starting ECP revealed no signs of recurrent or metastatic disease, or other abnormalities. S100 was monitored at 3-month intervals and remained with its normal range. An intriguing feature of ECP as an immunosuppressive therapy is its ability to control pathological T cell responses without compromising protective immunity.^11^ This apparently paradoxical effect is exploited in solid organ transplantation when minimizing conventional immunosuppression to treat opportunistic infections or post-transplant lymphoproliferative disorder.^12–14^ Moreover, when used in cutaneous T cell lymphoma, ECP enhances cytotoxic T cell (CTL) responses against tumour antigens, which is vital for its therapeutic effect.^15^ Priming of CD8^+^ T cells against viral and other tumour antigens is now a well-documented phenomenon after ECP, but its mechanism is not properly understood.^16,17^ It appears that monocytes present in ECP products become DC-like and acquire a capacity for cross-presentation of phagocytosed antigens to CD8^+^ T cells;^18^ however, it is not certain that such monocytes engraft in patients after reinfusion or whether they interact with T cells. As in other contexts, we speculate that ECP-treated monocytes deliver antigens and apoptotic signals to marginal zone DC and splenic red pulp macrophages, enhancing their cross-presenting ability.^19^ It is interesting to think that ECP might both suppress α-PD-1-induced inflammation and promote specific CTL responses.

## Data Availability

The data underlying this article will be shared on reasonable request to the corresponding author.
